# Data Mining and Statistical Approaches in Debris-Flow Susceptibility Modelling Using Airborne LiDAR Data

**DOI:** 10.3390/s19163451

**Published:** 2019-08-07

**Authors:** Usman Salihu Lay, Biswajeet Pradhan, Zainuddin Bin Md Yusoff, Ahmad Fikri Bin Abdallah, Jagannath Aryal, Hyuck-Jin Park

**Affiliations:** 1 Department of Civil Engineering, Faculty of Engineering, University Putra Malaysia (UPM), Serdang, Selangor 43400, Malaysia; 2 Department of Geography, Faculty of Environmental Sciences, Nasarawa State University Keffi (NSUK), Keffi 961101, Nigeria; 3 Centre for Advanced Modelling and Geospatial Information Systems (CAMGIS), Faculty of Engineering and IT, University of Technology Sydney, Building 11, Level 06, 81 Broadway, Sydney 2007, Australia; 4 Department of Energy and Mineral Resources Engineering, Choongmu-gwan, Sejong University, 209 Neungdong-ro, Gwangjin-gu, Seoul 05006, Korea; 5 Department of Biological Engineering, Faculty of Engineering, University Putra Malaysia (UPM), Serdang Selangor 43400, Malaysia; 6 Discipline of Geography and Spatial Sciences, School of Technology, Environments and Design, College of Sciences and Engineering, University of Tasmania, Hobart 7005, Australia

**Keywords:** debris flows, susceptibility, machine learning, MARS, SVR, LiDAR, GIS, remote sensing

## Abstract

Cameron Highland is a popular tourist hub in the mountainous area of Peninsular Malaysia. Most communities in this area suffer frequent incidence of debris flow, especially during monsoon seasons. Despite the loss of lives and properties recorded annually from debris flow, most studies in the region concentrate on landslides and flood susceptibilities. In this study, debris-flow susceptibility prediction was carried out using two data mining techniques; Multivariate Adaptive Regression Splines (MARS) and Support Vector Regression (SVR) models. The existing inventory of debris-flow events (640 points) were selected for training 70% (448) and validation 30% (192). Twelve conditioning factors namely; elevation, plan-curvature, slope angle, total curvature, slope aspect, Stream Transport Index (STI), profile curvature, roughness index, Stream Catchment Area (SCA), Stream Power Index (SPI), Topographic Wetness Index (TWI) and Topographic Position Index (TPI) were selected from Light Detection and Ranging (LiDAR)-derived Digital Elevation Model (DEM) data. Multi-collinearity was checked using Information Factor, Cramer’s V, and Gini Index to identify the relative importance of conditioning factors. The susceptibility models were produced and categorized into five classes; not-susceptible, low, moderate, high and very-high classes. Models performances were evaluated using success and prediction rates where the area under the curve (AUC) showed a higher performance of MARS (93% and 83%) over SVR (76% and 72%). The result of this study will be important in contingency hazards and risks management plans to reduce the loss of lives and properties in the area.

## 1. Introduction

Rapid population growth and its increasing concentration in urban areas have worsened the severity and impact of gravity-induced disasters across the globe, particularly in the mountainous environment. Debris flow is a natural hazard that occurs in a valley or on a mountain slope destroying everything it passes through. Debris flows occur when poorly sorted sediment, agitated and saturated with water, surge down-slopes due to the gravitational force, deposit on mountain bottom to form fans [1,2]. Iverson [3] defined debris flows as “turbulent flowing mixtures of sediment and liquid in nearly equal proportions”. Depending on the types of material involved, debris flows are variously known as mud spates, mudslides, mudflows, debris torrents, debris slides, debris floods, lahar and hyper-concentrated flows [2]. The ratios of solid and liquid constituents present in the mechanics of earth movements differentiate debris-flow events from other related phenomena. Accordingly, based on solid-liquid ratio contents one can easily categorize the type of events. In view of these, if solid sediment controls the liquid content, the constituent formed is avalanche; whereas if liquid forces control the solid content that results in floods, on the other hand, when an equal concert of liquid and solid presents it is formed as debris flows [3]. This forms a discern transition between avalanches and floods, based on their geological type and mechanical conduct characteristic [2]. In view of this, both solid and liquid (fluid) forces extremely influence the motion of debris flow, which could be responsible for its uniquely destructive power. Debris flow can be mobilized by either old or new landslides in the upper slopes that transport a mixture of equal quantity of grains (sediments) and fluid in high density verged downslope to a valley deposition zone, where the effects are usually felt on human economic development and social safety. Consequently, resulted in the loss of life and property. The event usually triggered by a number of factors such as heavy precipitation, lahar, earthquake, landslide and other anthropogenic activities [4,5,6,7,8,9,10]. Noteworthy, the prominent factors trigger debris-flow events in Malaysia is torrential rainfall that transforms landslides to a ribbon of surge liquid and solid sediments forming the debris flows [11]. Flows at high velocity along hill slopes and valleys exposing the regions to great risk of debris flow which results in a huge number of casualties and property damage along its path [8,12,13]; as the region suffers a frequent revisit period. A study by Berti et al. [14] estimated the reoccurrence periods for moderate debris flows to be a single occurrence in every two to three years interlude.

The hydrological factor of debris flow initiation (shallow slope failure, advancing erosion, activated by runoff), normally determines the action of runoff to precipitation of the unstable slope [15]. This event could be triggered by an influx of rapid concentration of fluid in the pebbly sinks that lack flora and soil; excessive overflow seepage rapidly transported at the sink channel where transient outlets incised into scree-covered slopes. Hence debris-flow events can be originated on such waterways at any sufficient runoff to activate free seepage solid grains of the channel bed [15,16]. So also, debris flow activated from landslide is driven once the triggering rainfall produces an upsurge of the free-water present in soil related grains with detailed groundwater level travels over its bed. Details on debris flow activated from landslides can be found in [15,17,18,19,20,21]. The occurrences of debris-flow triggering factors are expected to intensify due to climate change [22,23,24,25]. Other studies have established the increasing influence of climate change on both magnitude, the intensity and the occurrence of debris-flow disaster [20,26,27]. 

There is a strong connection between local intensity rainfall rate and debris flow initiation in other locations around the globe [28,29,30,31]. The debris flow literature is also replete with studies that incorporate estimates of rainfall intensity in space and time to debris flow initiation [16,17,32]. The studies rely on surface-based rainfall observations near debris source region and proven effective in identifying rainfall intensity threshold for mobilizing debris in regions prone to mass-wasting [29]. Nakatani et al. [10] investigated the debris flow disaster scenario caused by severe precipitation in a stream of Kiyomizu (Kyoto, Japan). Nakatani and his co-researchers developed a Hyper KANAKO 2D system based on a numerical model to simulate debris flows using rainfall intensity, archived landform data, DEM and digital surface (DSM). They observed that a small mesh size of a terrain feature is capable of triggering debris flow over appreciable distance.

In another paper, Lari et al. [33] reported debris flow risk assessment at a regional scale for an Alpine valley. The study observed the impact of debris flow over a period of three decades, particularly in the central Alpines, Northern Italy. It reported that between 1983 and 2004, debris flow affected about 1000 km^2^ with 31 casualties recorded and over 90,000 people rendered homeless. 

Developing countries suffer more from natural disasters. In Malaysia, debris-flow disaster was first recorded in May 1961 in Pahang; since then, debris-flow events have been claiming lives and properties worth of thousands of Ringgits across the country. Jamaludin et al. [34] identified eight locations of active debris flow events in Peninsular Malaysia. They include Genting Sempah Selangor (km 39 of Kuala Lumpur—Karak highway) on 30 June 1995; Johor (Gunung Pulai) on 28 December 2001; Selangor (Kuala Kubu Baru-Gap road) on 10 November 2003; Perak Gunung Tempurung (km 302 North-South expressway) on 10 November 2004; Lentang in Pahang (km 52 of Kuala Lumpur-Karak Highway) on 2nd November 2004; Perak (km 33 Simpang Pulai-Cameron Highlands Road) on 12 April 2006; Pahang (km 4 to 5 Fraser’s Hill Road) on 15 November 2007; and Kelantan (Lojing-Gua Musang Road) on 3rd January 2009. In a review paper, Abdul Rahman et al. [35] outlined areas of major landslides induced debris flow disasters in Peninsular Malaysia and of particular interest are the Cameron Highlands mud flood of 2013, Hulu Langat landslide in 2011, and Highland tower collapsed in 1993. Over time, about 21,000 landslides have been documented across Malaysia. About 16,000 (76%) of the documented events occurred in Peninsular while 3000 and 2000 cases feature in Sabah and Sarawak, respectively [36]. The report has also shown that within 18 years (1993 to 2011) about 28 major landslides occurred in Malaysia with over 100 casualties recorded [36]. From 1973 to 2007, the worth of property loss from landslides related disasters was estimated at about a billion US dollars. For example, the collapsed of 14-storey building of the Highland Tower in Ulu Klang, Selangor, alone took 48 lives [36]. It was observed that aside rainstorm, poor slope management; human activities were the major factors responsible for the frequent landslides/debris-flow events in most of the regions in Malaysia. Due to the threat posed by increasing frequency of local rainfall intensity, landslide occurrence in the region, there is a need to better understand the area susceptible to debris flow and identify the suitable methods for prediction. 

Laser scanning technologies (airborne and terrestrial) have been widely utilized and described suitable for landslide hazard mapping in forested areas [10,25,37,38,39,40,41,42,43,44,45,46]. In Peninsular Malaysia, LiDAR technology has been used for landslide hazard mapping, modeling and other related phenomena e.g., [6,47,48,49,50,51,52]. However, only very few studies have been carried out on debris flow susceptibility mapping using LiDAR data in this region e.g., [11,47,53,54,55,56,57]. LiDAR DEM dataset allows retrieving primary and secondary landslides conditioning factors to predict susceptible and hazard zones.

The aim of this study is to identify areas susceptible to the debris flow in a typical tropical region with the thickly forested environment using different Machine Learning Algorithms (MLAs). For debris flow detection and susceptibility analysis, Elkadiri et al. [38] outlined three procedures (deterministic, heuristic and statistical models). Deterministic models are established on mathematical associations that obeys semantic rules that trigger a mass movement, heuristic models are associated with the weight of the individual conditioning factors based on expert opinion [58], and statistical models are data-focused. The statistical-based models rely on either the influence of single (bivariate statistics) or multiple (multivariate statistics) conditioning factors [48,59,60,61]. In this study, the machine learning approach (advanced statistics) model was employed to assess debris flow susceptible areas in the study site. In this study, we employed MARS and R-SVM algorithms to prepare debris-flow susceptibility zones. To the best of our knowledge, no existing research on debris-flow susceptibility mapping has specifically utilized these models (MARS and R-SVM).

## 2. Research Background

Recently, laser scanning technology, often known as Light Detection And Ranging (LiDAR) systems, has become widely accepted as a breakthrough in remote sensing surveying methods since 1999 [62]. This system is used to identify and detect attributes of terrain surface. The architectural and structural development of LiDAR system operations for prosperous data acquisition have been reported in the literature [43,45,62,63,64,65,66,67]. Based on the platforms, three types of laser scanning technologies have been reported, namely; Airborne Laser Scanning (ALS), Terrestrial Laser Scanning (TLS) and Mobile Laser Scanning (MLS). Their components and mode of operation in data capturing differs; therefore, in this research we considered ALS system because it allows wider area coverage than TLS and MLS. All LiDAR data yields favorable fine-resolution geomorphometric and topographic data in 3D that can hold significantly to an accurate surface representation with a very-high spatial-resolution over a wide area [68]. The LiDAR data reveals micro-morphological features, which are significant for initiating debris flow even under dense vegetation canopies and demonstrates capability in observing earth movements. The proficiency of the LiDAR system has made it valuable in detecting and mapping some catastrophes related to earth movements. The tropical climate of Malaysia makes debris flow analysis a difficult task. Tree canopies, dense forests and rugged terrains constitute a barrier to derive complex morphological and topographic features from microwave remote sensing data [40,44,46,69,70,71]. Unlike Synthetic Aperture Radar (SAR) data derived elevation model, LiDAR data is not affected by topographic shadowing because of the angle of inclination (<20° off-nadir) of LiDAR sensors [11,44,45,48,64,72,73]. In a recent study, a LiDAR-derived Digital Elevation Model (DEM) was considered the most favorab le terrain data used to derive topographic attributes integrated with appropriate modelling approach to detect and predict environmental disaster [67,74]. LiDAR-derived DEM provides fine spatial-resolution topographic data, which accurately depicts the terrain configuration [70,75,76]. 

In related literature, a number of approaches have emerged for debris-flow modelling on run-out and its trajectory. Debris-flow run-out models include physical approaches, empirical approaches and dynamic approaches, although run-out and trajectory are not covered in this paper. Recent studies have shown that machine-learning techniques are very effective for assessment of geo-hazard related disasters [6,52,77,78,79,80]. A number of MLA have been developed and adopted in various areas of geosciences. Amongst a few are multivariate adaptive regression spline (MARS) [6,81,82,83,84,85,86,87,88], random forest (RF) [6,42,69,80,89,90,91] and artificial neural network (ANN) [47,49,52,92,93,94,95,96,97,98,99,100]. Data over fit and large data requirements are among the drawbacks associated with the conventional MLA. Advance MLAs have edge over the conventional approach in overcoming their challenges. This motivates the utilization of some associated approach of advance MLAs such as Regression Support vector machine (R-SVM) that is used in other remote sensing related applications [6,51,77,78,80,89,99,101,102].

In some papers, different types of models have been compared in various types of geoscience application. For example SVM and random space in [78,99,103], ANN-fuzzy logic approach [48,61,81,98,104,105]; Decision tree and Neural Networks [77,92,98,105,106] have been applied elsewhere. Reports have shown that some of these approaches out-performed the conventional techniques [49,107,108]. Although every modelling approach has its related merits and demerits which underpin their guiding assumptions, individual studies using MLA for debris flow susceptibility modelling are limited, especially in the case of the newest algorithms in the field of geo-hazard such MARSpline. In the instance of SVMs, parameterization needs or operability values have not been thoroughly investigated; although the few identified approaches are usually adopted for hazard mapping especially landslide susceptibility. Conversely, to our best knowledge, no existing research have compared the two approaches using LiDAR-derived DEM to assess and predict area susceptible to debris flow.

However, to the best of our knowledge in the literature search, very few studies have employed machine learning approach in debris flow susceptibility mapping; Some good examples are the combination of weight and extension method [109] and Analytical Hierarchy Process (AHP) [109,110,111,112]. Some of the studies on debris flow susceptibility in Malaysia [113] have adopted Horton open-source Flow-R software for the identification of debris-flow source area. However, the Horton (Flo-R) method lacks flexibility in the selection of the conditioning factors used and was developed for a regional evaluation purpose [33,114,115].

MARSpline technique is a non-parametric MLA and non-linear estimation procedure. This approach adopts discretization regression gradients at diverse interludes data frame for model assessments [116]. The MARS is best at identifying optimal variable connections and transformations; expose intricate hidden data models successfully, often on a high scale. Also, the model has revealed significant data relations and pattern, that are hard or even awkwardly attained by other approaches [117,118,119]. The basic advantage of MARS approach compare to other MLAs is its ability to simplify the challenges of a nonlinear relationship between dependent and explanatory variables. As an alternative, MARS structures a relation from established basis functions (BF) and coefficients, which are generally determined from the regression information (www.statsoft.com). Certainly, the areas in which this model have been applied include landslides [11,31,42,82,83,90,116,120,121,122,123,124,125,126]. The superiority of MARSpline over some MLAs have been demonstrated in other fields of research [81,125,127,128,129,130].

Meanwhile, the second model lies in the principle of SVM (R-SVM) approach; established on statistical learning concept. The model was adopted due to the intricacies of procedures in geosciences where the relationships among different data types are usually non-linear. Therefore, it was is recommended to employ R-SVM in a situation of mild nonlinearity; sometimes validation and reduction in data dimension can be handled in the SVM algorithm. The approach processing or runs time is smaller compared to other data-mining algorithms (neural network). Various researchers have adopted this MLAs separately [42,90,99,122]. 

## 3. Materials and Methods 

**Figure 1:**
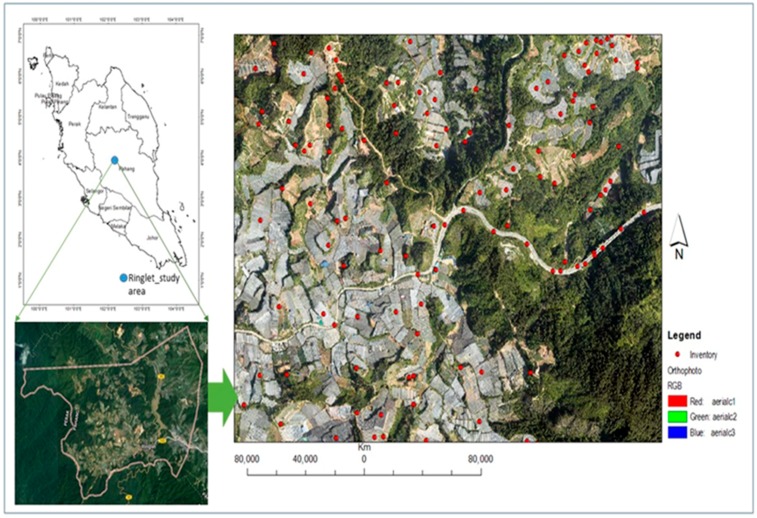
The Study area map.

This study is conducted in Cameron Highlands, around the Ringlet area of Pahang in Peninsular Malaysia. Geographically, the study area lies within latitude 4°22′52″ N and 4°25′48″ N and longitude 101°22′30″ E to 101°23′30″ E (Figure 1) covering an area of about 25 km^2^. This area is specifically chosen because of the frequent occurrences of landslides and slope instability that often results in debris-flow [47,79]. In terms of physical development, the study area experiences rapid developments, which have greatly altered the natural slope, potentially, putting the community living in downslope in danger. Generally, the topography of the Cameron Highlands area is underlain by rising and falling plateau ranging between the elevation of 1000 m and 1676 m a.s.l. [47]. The geological formation comprises of granites of various categories of megacrystic biotite and associated micro-granite (muscovite) [48,55,131] and meta-sediments comprising of slate, (Sedimentary rock) phyllite, limestone, sandstone and schist (metamorphic rock) [61]. The mean annual rainfall records range from 2500 to 3000 mm per annum. There are two raining seasons in the study area, which include; the September to December regime and the February to May regime but the peaks are recorded between March to May and November to December respectively. During the peak period, rainfall of between 88 mm to 100 mm in a single day usually triggers landslides [6,11,50,52,55]. When the local rainfall rate exceeds a distinctive intensity threshold, a runoff-generated debris flow may likely be initiated.

The occurrence of debris-flow events in this region of complex terrain is strongly induced by ubiquitous slopes-bed deposits of weathered-source grain sediments and weather system. Such as recurrent monsoon in the wet season is associated with thunderstorms that result in a locally intensive downpour; the recorded rainfall intensity is sufficient to trigger debris flow [17,20,26]. The risk presented by debris flow is dynamic and the threat related to a hydrological hazard could aggravate severe hazard in the near future due to modifying climate pattern in the tropical region that could change the precipitation system.

### 3.1. Data, Software and Techniques 

**Figure 2:**
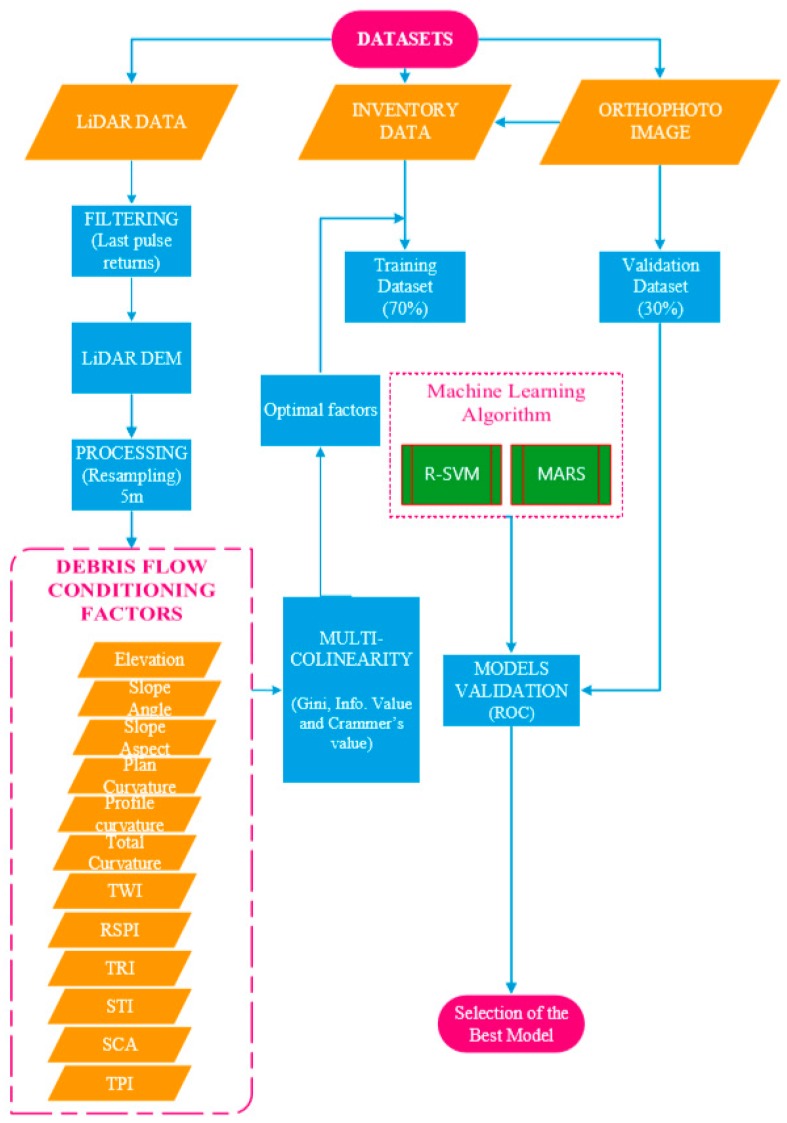
Overall methodological workflow.

The systematic procedures utilized to achieve the objectives of this research paper is summarized and illustrated in Figure 2. The datasets used in this study are ALS data, orthophoto and landslide-inventory data. The site was visited twice in 2017, first between 15th and 18th May and later from 19th to 23rd December. Twelve conditioning factors comprising of topographic and geomorphic parameters were derived from 5 m resolution LiDAR-derived DEM to model debris flow event using SVM and MAR Splines algorithms. The inventory data was assigned to the dependent variables while the conditioning factors represent the independent variables. Data processing was accomplished using different software packages these include Whitebox (open source software; https://www.whiteboxsoft.com), ArcGIS 10.6 (ESRI, Kuala Lumpur, Malaysia) software environment, LAStool in ArcGIS, Salford predictive Modeler 8.1 (Salford System, San Diego, CA USA), Statistica (StatSoft,Tulsa, OK, USA) and Waikato Environment for Knowledge Analysis (WEKA) version 3.9 (University of Waikato, Halmilton, New-Zealand).

### 3.2. LiDAR Data Acquisition and Preparation

Airborne LiDAR data was acquired with discrete pulse return systems over Cameron Highlands in support to a regional scale assessment of landslides [11,52,132]. The LiDAR database comprises of pulses (beam), gathered in the scope of PBRC Project, 10th Malaysia Plan (RMKe-10), through Minerals and Geoscience Department Malaysia (JMG) in the year 2015. The LiDAR data was captured on the 15 January 2015 by a Malaysia-based Company, using a scanner aboard EC-120 Helicopter flown at an average altitude of 1676 m above m.s.l. The sensor was calibrated to sample at a 25,000 Hz pulse rate that yields ground point density of 8 points/m^2^, 0.5 point spacing with a vertical accuracy of 15 cm on vegetated terrain and horizontal accuracy of 25 cm, fall within the acceptable threshold value for LiDAR processes in the area of land formations [133]. The acquisition exercise, footage stored a number of pulse-returns together with the first and last returns. In addition, an orthophoto image (0.09 × 0.09 m) was obtained during the LiDAR campaign, (Figure 1) in the scanned area using EOS5D Mark III camera with a focal length of 35 mm mounted on the aircraft. The camera has a horizontal and vertical resolution of 72 dpi, respectively, and exposure time of 1/2500 s. 

The data pre-processing task commenced with a subset of the area of interest that covers a span of (5 km × 5 km) 25 km^2^. LAStool of ArcGIS software package was utilized to filter out the other returns (vegetation and building) owed to their insignificance to this study. Keeping single LiDAR point cloud records size representing the ground surface, which is of interest to this research. First and last pulse return that made-up point cloud using morphological filtered approach to separate ground points from non-ground points, the latter represented the surface terrain morphology of the area and is selected for further analysis. The statistical reports have shown the LiDAR total point return is 3,998,752 where 2,848,914 (71.25%) was classified ground and 1,149,838 (28.75%) non-ground using LAStools add-in in ArcGIS software. The filtered ground LiDAR return points cloud dataset was interpolated into raster grids using a natural neighbor algorithm (NNA) via Triangulated Irregular Network (TIN) to produce a digital terrain model (DTM) of 5 m cell size utilizing the ArcGIS 10.6 software tool. Natural neighbor presented a ubiquitously smooth surface, but the position sample differs. This was chosen because of Baker and Coop [134], who stated that no technique of interpolation is superior to another when used for the interpolation of a DTM; thus they observed the model conservative and has the lowest error term. NNA adapted the principle of autocorrelation to the added inverse distance to a power function and related weights to the input samples in proportional areas to interpolate a value. Hence, the LiDAR data was used to produce a DEM of the study area at 5 m resolution. The LiDAR-derived DEM derived defines elevation of surface in term of planes, valleys and crests. 

Obviously, no ideal grid resolution exists, but rather a range of suitable resolution [133] despite its significant role-plays in terrain efficiency mapping. Selection can be optimized to a certain level to satisfy both processing capabilities and representation of spatial variability. A grid resolution that optimally represents diverse terrain surface is required that is capable to reflect the majority of geomorphic attributes. A finer grid resolution can introduce local artefacts and slow down computation of terrain parameters. Kienzle [135,136] pointed a systematic overview of the effects of various grid resolutions on the reliability of terrain parameters suggesting fine grid resolutions from 5 m to 10 m. A LiDAR-derived DEM was generated at 5 m cell size signifying the LiDAR post spacing [135,137], exhibit hydromorphic structures used to generate the debris-flow conditioning factors. 

Very high-resolution offers the prospect of the greater reliability of topographic features, which improves the identification of debris flow. The topographic reflectance dataset was utilized to generate primary and secondary terrain surface derivatives. Nevertheless, it could produce inherent issues related to the surface; generate anisotropic effects in the spatial resolution, generating artefacts. Pits in the DEM were filled to sieve out undulating artefacts to attain hydrological connectivity [138]. For computational efficiency, lessen prediction errors and increase model accuracy, the original DEM 0.5 m resolution data was spatially resampled to 5 m subsequently. According to Keijsers et al. [139], fine DEM resolution represents geomorphic and topographic configurations better than coarse DEM. Research has demonstrated the effects of different DEM resolutions on susceptibility mapping which indicates low DEMs resolution hinders true representation of terrain attributes because it smoothens local surface variability [139]. Based on the assumption that terrain conditions that initiate an event in the past will likely lead to a reoccurrence of such event in future, the LiDAR DEM was used to derive both the primary and secondary conditioning factors utilized in this investigation. 

Statistical analysis of the debris flow inventory data and its conditioning factors were combined to formulate an independent input variable utilized to predict area susceptible to debris flow. This allows assessment of areas prone to debris flow for emergency planning. Researchers [70,71,77,85,99,107,115,140,141,142,143,144,145,146,147] have identified the relationships between earth movements and the conditioning factors associated with morphological, topographical, climatological, hydrological and human activities. However, the accuracy of debris-flow susceptibility mapping depends on the high quality of these input model factors data.

Unlike land use and climate variables which change rapidly over a short time, lithology and soil properties were all not considered as part of the conditioning factors in this study because they are assumed to be constant over a longer time scale [139]. In addition, rainfall data was exempted from the model because it is considerably uniform [47,148] over the small area.

#### 3.2.1. Derivation of Conditioning Factors

**Figure 3:**
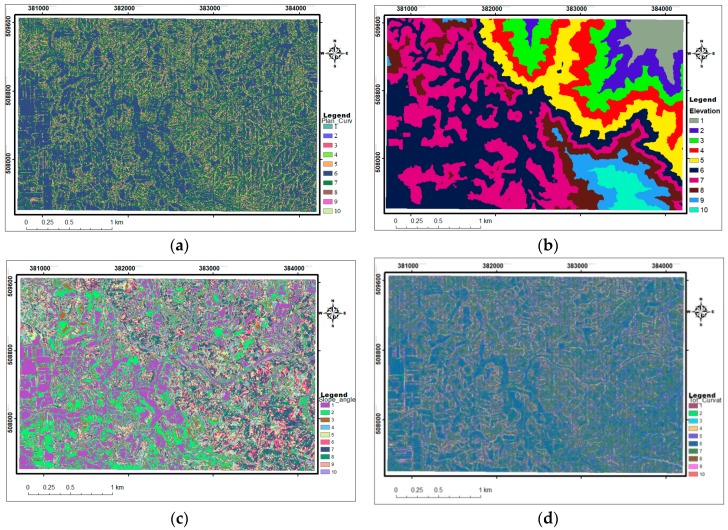

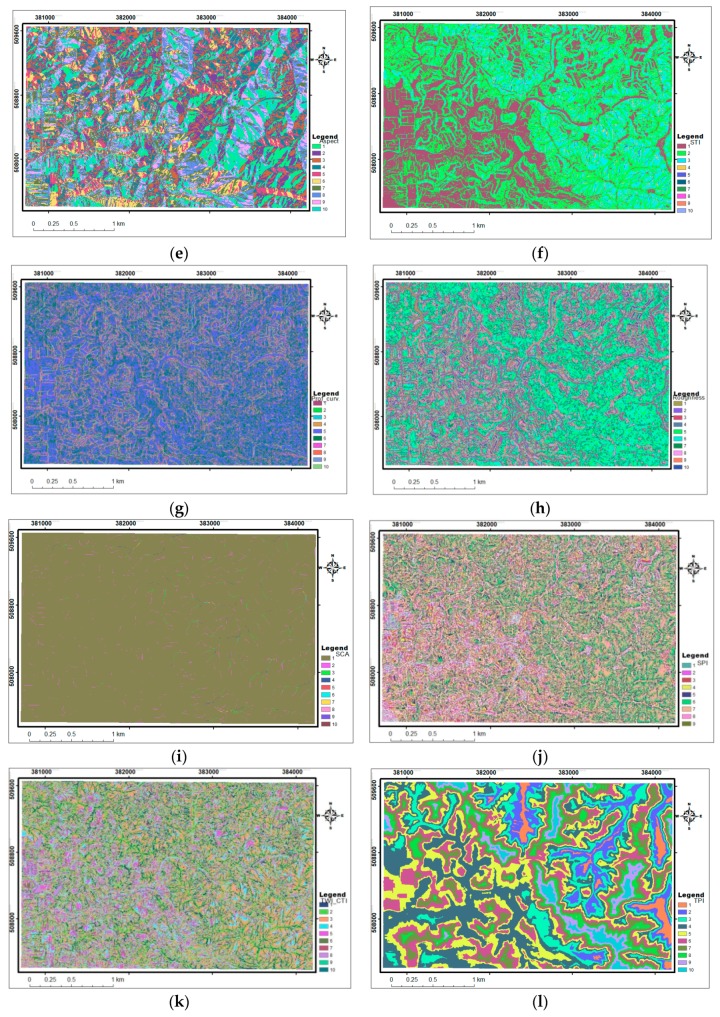
Cont. (**a**). Elevation, (**b**). Plan curvature, (**c**). Slope angle, (**d**). Total curvature, (**e**). slope aspect, (**f**). Sediment Transport Index (STI), (**g**). Profile Curvature, (**h**). Roughness index, (**i**). Stream Catchment Area (SCA), (**j**). Stream Power Index (SPI), (**k**). Topographic Wetness Index (TW) and (**l**). Topographic Position Index.

From, the thorough review of relevant literatures e.g., [38,60,115,149,150,151,152,153,154,155] twelve conditioning factors were selected and they were derived from the 2 m LiDAR DEM. These factors include; elevation (Figure 3a), plane curvature (Figure 3b), slope angle (Figure 3c), total curvature (Figure 3d), slope aspect (Figure 3e), Sediment Transport Index (STI) (Figure 3f), topographic profile curvature (Figure 3g), Topographic Roughness Index (TRI) (Figure 3h), flow accumulation/SCA (Figure 3i), Stream Power Index (SPI) (Figure 3j), Topographic Wetness Index (TWI) (Figure 3k), and Topographic Position Index (TPI) (Figure 3l). Generally, these variables are critical factors that control the occurrence and characteristics of mass movement and are widely adopted in geo-hazard modelling and assessments e.g., [7,23,38,39,42,48,81,89,156].

The elevation factor was extracted from LiDAR-derived DEM, which describes the general configuration of topography. It provides height value for each point on the surface relative to the reference datum, in this case, the mean sea level. Area of low altitude collects surface runoff rapidly from the slope angle, which allows debris deposition. High elevation initiated debris, flow on varying slopes and angle. The elevation was reclassified into five classes using the natural break classification scheme e.g., [85,87,98,110,122].

Burrough and McDonnell [157,158] recommended Horn slope algorithm to be more appropriate to estimate slope angle utilizing remotely sensed LiDAR-derived DEMs on rugged terrain; most importantly this algorithm is embedded in ArcGIS software (ESRI). Hence, slope angle was determined using the surface analysis tool of spatial analyst extension in ArcGIS 10.6 software environment, using elevation as input data. This factor is a measure of a tangential surface robustly potential proxy for debris flow occurrence. Thus, slope gradient or angle is one of the core conditioning factors in debris flow mapping and modelling [11,115,153,159,160] and terrain morphology. Geomorphometric applications are effectively determined on the local slope, calculated in the direction of undue rapid gradient from discrete pixel to its diagonal or adjoining grid cells. Different activities take place at different slope range, for example, at slope angles between 15° to 35°, debris flow initiation occurs [27,102,113,160,161,162,163,164] whereas run-out and deposition mechanism occurs at a slope between 15° to 20° and <9°, respectively [12]. 

Another slope related parameter is the slope aspect. Slope aspect was computed with surface analysis tool via spatial analyst tool of ArcGIS 10.6 software environment. The output was converted from radians to degrees [165], the values range from 359° to 1° representing a cardinality (direction) of the surface. While total curvature depicts the convex or concave terrain of neighboring grids, aspect describes the direction, which reveals a surrogate for exposure to the climatic factors. This shows the horizontal direction of the slope of a mountain orientation [77], which activates weathered and eroded materials to be transported down the slope in the study area. As earlier discussed, most of the past debris-flow hazards in the area were triggered by landslides (new or old) combined with heavy downpour (duration and intensity) during the monsoon seasons, with a defined direction of wind flow [166,167]. For example, during the monsoon season, the wind flows in Southeastern and Northeast directions [166], noteworthy to mention that the NE monsoon wind direction and movement bring out heavy rainfall while the SE monsoon is a dry wind. This heavy downpour triggers flooding and landslides. The slope aspect was expressed in a unit degree (0°–360°), oriented clockwise directions starting from the north position, The facet of the terrain defines the effects of weathering and the amount of rainfall in the area based on the level of the exposed surface [77].

TPI is a degree of association between the elevations of a principal position to the elevation of adjacent terrain (nearest neighbor), describing planes trench, and crests. TPI was estimated following the methods of [168,169] focal statistic tool was used with radius neighbourhood shape, maximum and minimum kernel window cell size (3 × 3) and (200 × 200) [165].

The flow accumulation parameter defined the drainage pattern of the study area. The parameter presents the cumulative amount of the number of pixels that logically drain into outlets [38]. The collective hydrologic flow value signifies the number of input pixels that contribute to the channel. To avoid misleading result, sinks in the DEM was filled before it is used to produce flow direction and subsequently flow accumulation. 

Some debris-flow conditioning factors (Total, profile and plan curvatures) showed a positive relationship with flow accumulation. Profile curvature was estimated using the slope simplification approach revised by [170]. The profile curvature defines two distinct characteristics of the topography, convexity (positive) and concavity (negative) curvature of the terrain surface [77,142]. The concave curvature accumulate water and exhibit convergent of soil and hold deposit sometimes indicating positive pixel value on an image. While convex type of curvature does not hold any earth material and indicated by negative values and a flat surface with a zero value.

In addition to the parameters discussed above, a number of morphometric related indices were also calculated and included in the modelling. First is the Topographic Wetness Index (*TWI*) which involves the upslope contributing area, a slope raster and a number of geometric functions [171,172]. It is important to consider carefully the flow accumulation and slope because how they are calculated will strongly affect the *TWI* values. The value of each cell in the output raster is the value for the flow accumulation raster for the corresponding DEM. *TWI* is mathematically expressed as [90]:

(1) $$TWI=ln\left[\frac{As}{tan\left(\beta \right)\;}\right]$$

where *ln* is the natural logarithm, *As* is the flow accumulation and β is the slope angle. 

Stream Power Index (*SPI*) as a hydrological factor is a product of catchment area and slope. *SPI* is used to identify suitable locations for soil conservation measures to reduce the effect of concentrated surface runoff [17,20,87]. It has a considerable impact on debris flow development which indicates the potential of a channel to cause erosion [77]. *SPI* is generated using Equation (2):

(2) $$SPI=\frac{As}{tan\left(\beta \right)}$$

Sediment Transport Index (*STI*) accounts for the effect of topography on erosion. The 2D catchment area is used instead of the 1D slope length factor as in the Universal Soil Loss Equation:

(3) $$STI={\left[\frac{As}{tan\left(\beta \right)}\right]}^{22.31/0.6}$$

(4) $$Z_{i,n}=\frac{Z_{i}-\left(\frac{Z_{max}}{2}+\frac{Z_{min}}{2}\right)}{\frac{Z_{max-}Z_{min}}{2}}$$

where $\;Z_{i,n}$, represent the standardized value for $\;Z_{i}$, which signifies separate data point, $Z_{min}$ and $Z_{max}$ are the minimum and maximum in the datasets.

In data pre-preprocessing stage, we normalized individual datasets which transform the factor values to a common standard dimensional level. Because of the large variability observed in the range values of the processed conditioning factors. Each conditioning factors was normalized using (Equation (4)) that produced a pixel value ranging between 0 and 1, purposely to unify the conditioning factors, reduce high dimensionality differences in the modelling task [38], also allowing relative estimate and combination. Parameter values standardization can improve the model response, action that tends to stabilize variance, remove nonlinearity, and counter non-normality (SAS tutorial, 2019) and leads to better model fits. 

#### 3.2.2. Inventory Data for Model Training and Validation

Inventory data is vital to debris flow susceptibility analysis [6]. Based on existing historical records, 320-point locations of previous landslides/debris-flow events were obtained. The validity of the points was verified during the site visit using handheld GPS (GERMIN GPSMAP 78S) and the information provided by the local residents. Another 320 points representing non-debris-flow locations were randomly selected and combined with the inventory data for the modelling. The 640 points were labelled 1′s and 0′s for the classification process where the former indicates the presence of debris flow (in the past) and the latter represent the absence of debris flow [55]. For each point location, corresponding cell values of the normalized conditioning factor datasets were extracted using multi-values to point tools in ArcGIS 10.6 software and randomly split into training and test datasets using ratio 70% (448) to 30% (192) to develop and validate the resulting debris flow susceptibility model [38,55,78,104,111,112,115,154,173,174,175]. As earlier mentioned, MARS and SVR data mining techniques were adopted and these MLAs often accept binary classifications [107]. 

### 3.3. Debris Flow Susceptibility Modeling 

Data pre-processing carried out to select relevant factors that have no influence on others but have strong relationships with the conditioning factors using the correlation approach and explores the factors accuracy metrics information value (IV) and Gini coefficient. Machine learning approaches of SVM and MARS are evaluators considered in debris flow susceptibility mapping.

#### 3.3.1. Debris Flow Conditioning Factor Selection 

Several debris-flow conditioning factors have been experimented in many studies as input into data-mining algorithms. However, the selection process of the parameters is mostly not given proper consideration [176]. The inclusion of input parameters into modelling algorithms using the expert or a priori knowledge of the phenomenon could affect the quality of the model output [101,176]. The quality of a model’s output does not necessarily depend on the number of input parameters. Studies have established that the use of a huge number of factors practically increases the cost of data collection and processing time in addition to the possibility of generating misleading regression estimate [6]. Therefore, testing the credibility of input parameters by optimizing the conditioning factors is essential for reliable debris flow susceptibility modelling.

A very useful concept of variable selection in debris flow susceptibility model was done by optimizing information value (IV), utilizing Gini and Crammer values [42] under spearman correlation to examine the multi-collinearity effects in the predictor (input variables). This step allows identifying and subsequently discarding unrelated and out of range dataset, that usually affects the predictive accuracy of the model. It has been reported that a good feature subset is one that contains features highly correlated with the target, yet uncorrelated with each other [177]. IV (Equation (5)) is a statistical method for feature prediction that removes insignificant factors at 95% confidence level. The predictive power is expressed as such; <0.02 (bad predictor), 0.02 to 0.1 (weak predictor), 0.1 to 0.3 (moderate predictor), 0.3 to 0.5 (strong predictor) and >0.5 (Excellent predictor), which can also be expressed in percentage (%). Usually, variable all within moderate and strong predictive power are selected for model building. Optimum conditioning factors were selected using the *IV*, mathematically expressed as:

(5) $$IV=\left[\overset{k}{\underset{i=1}{\sum }}\left(g_{i}-b_{i}\right)\;\times \;ln\left(\frac{g_{i}}{b_{i}}\right)\right]\;\times \;\%$$

where k is the class size of individual conditioning factors, $g_{i}$ represent the distribution of good and $b_{i}$ denote the distribution of bad is the column-wise in percentage distribution of the total “debris flow” circumstances in the ith case, $b_{i}$ is the percentage distribution of the total “no-debris flow” circumstances. The Gramer’s *V* statistic and Gini coefficient were calculated on individual conditioning factor. Gini coefficient is a measure of inequality in a given population and has an index value ranges from 0 (all factors are equal) to 1 (strength of affinity in an infinite variable). In addition, the Cramer’s *V* quantify the relationship amongst the conditioning factors and also, its value range from 0 (no relationship) to 1 (perfect relationship). The Cramer’s *V* is expressed as [92,116]:

(6) $$V=\sqrt{\frac{x^{2}}{n\;\times \;min\left(w-1,k-1\right)}}$$

where $x^{2}$ is the *Chi-square*, n is the number of entries existing in the dataset, w is the size of the categorical dependent variable (i.e., “non-debris flow” and “debris flow” (=2) and  k is the size of factors present in the explanatory variables (i.e., the 12 debris flow conditioning factors).

Since the conditioning factors are non-parametric datasets, we adopted multiple regression using Spearman correlation method to further evaluate the relationships between the predictor and the conditioning factors (i.e., check for multicollinearity effects) purposely to indicate the level of insignificant relationship between the conditioning factors. Finally, all the conditioning factors that meet this hypothesis were selected and included in the modeling. Some of the input factors with a risen noise negatively contribute to the model building.

#### 3.3.2. Multivariate Adaptive Regression Splines (MARS)

Multivariate Adaptive Splines (MARS) first put forward by [178] as one of the novel artificial intelligent approaches. MARS is a flexible, swift and accurate model for predicting continuous binary effects [116]. This approach integrates the mathematical structure of splines and the classical linear regression. The binary recursive splitting and instinctive search algorithm make it a model which is capable of predicting values from a given target variable (continuous and categorical) obtain from a dataset (independent variables). MARS employs basis function (BF) (Figure 3), a non-parametric and non-linear procedure that adopts discretization regression gradients at diverse intervals of the data frame for estimations. De-Andres et al. [116] noted that the recursive segregating approach that controls generalized additive modelling (GAM), classification and regression tree (CART) jointly motivates the MARS technique. The elasticity of the MARS method makes it possible to model correlations that embrace collaboration with limited parameters [116]. MARS is best at identifying optimal variable connections and transformations that expose intricate hidden data models successfully. It is also the capability of revealing a pattern that is rarely possible for other approaches to achieve [117]. 

The principal gain of the MARS model is that it clarifies the difficult in a nonlinear relationship between dependent and independent variables [126]. To do this, it approaches the underlying function through a set of piecewise functions, basic functions (BF), also known as Splines Polynomials [128,130]. The BF represents the information contained in one or more independent variables and was selected through a systematic process. The MARS model  f(x) is developed as a linear combination of BFs and the connections between them. MARS model is express in (Equation (7)) as:

(7) $$y=f\left(x\right)=\beta_{0}+\overset{M}{\underset{m=1}{\sum }}\beta_{m}h_{m}\left(x\right)$$

where *y* is the model expected value through a function  f(x) which could be disintegrated into an initial constant $\;\beta_{0}$, $\beta_{m}$ is the coefficient of the *mth BF*, $h_{m}\left(x\right)$ is a *BF*, and n is the number of *BFs* in the model [88,179]. BFs are functions that proceed in either twofold modified formula as [90,122]:

(8) (0,x−α) or  (0,α−x)

where *x* is an independent (0 factors) variable and α is a constant, represent the node-to-node position. MARS utilizes truncated power functions as spline basis functions [180]. The Dual contiguous splines overlap at the knot to preserve the occurrence of the *BF* [122,127].

To generate the final MARS model, first, the forward stepwise algorithm (Equation (7)) was employed where a number of successive pairs of *BFs* of the model are added to obtain optimal performance [128,178]. This algorithm introduces complex over-fittings, which usually produce a poor estimate of the predicted results [116]. So, a second backward stepwise algorithm was applied to resolve the over-fitting by removing redundant *BFs* from the MARS model using Generalized Cross-Validation (GCV) (Equation (9)) [134]. The GCV limits the number of BFs to be included in the performance [128,130,179,181,182,183]:

(9) $$GCV=\frac{\frac{1}{N}\sum_{i=1}^{N}{\left[y_{i}-\hat{f}\left(x_{i}\right)\;\right]}^{2}}{{\left[1-\frac{C\left(B\right)}{N}\right]}^{2}}$$

where *N* is the number of data $\hat{f}\left(x_{i}\right)\;$ is the projected value of the MARS model and C(B) is a complexity penalty that rises with the number of basis functions (BF) in the model. C(B) is obtained from Equation (10):

(10) C(B)=(B+1)+dB

where d, a penalty is for each BFs included in the model as a smoothing factor, and B is the number of basis functions in Equation (7). The penalizing parameter (d) signifies a cost BF optimization [128]. Increasing the number of *d* influences the size of the knotss while assigning higher values for *d* allows the use of less number of knots, smoother function estimates and small BFs [116]. The optimal value for the penalizing factor is between  2≤d≤4; however, Friedman [178] recommends 3. In the current work, a value of 3 is assigned to the penalty factor *d* similar to Zhang et al. [128]. In addition, the maximum interaction BFs level is limited to 10 for MARS approach with the optimal model size $M^{*}$ of 2 [130].

The MARS model was also used in the optimal feature selection process [26,81]. The contribution of each conditioning factors in the debris flow model was assessed in ANOVA. This procedure involves all the BFs related to both the individual factor and pairwise interaction aggregated separately through analysis of variance decomposition for statistical significance [178,183]. This was achieved by evaluating the coefficient of determination $R^{2}$, the coefficient of correlation r, mean average error (MAE), root mean squared error (RMSE), performance index ρ and receiver operative curve (ROC). Finally, the performance rate and index are compared with SVM. 

#### 3.3.3. Support Vector Machine (SVM)

Support vector machines (SVM) is a supervised machine learning technique developed by Vladimir Vapnik and his colleagues in 1995 at AT&T Bell Laboratory [184]. SVM was originally developed for classification (C-SVM) and later adopted to solve regression (R-SVM) problems called support vector regression (R-SVM) [185,186]. The algorithm is based on statistical learning theory and structural risk minimization capable of addressing linear and non-linear multivariate regression problem [187]. SVM separates two opposite analogous hyperplanes and get the best separating distance between two vectors [106,127]. If the space between them is large, then the overall error of the classifier is reduced. The relationships among different categories of data in geosciences are mostly nonlinear. Therefore, it is recommended to adopt C-SVM also in nonlinear problems. Details on the SVM algorithm can be found in [102,105,188]. Mathematically, R-SVM is expressed as:

(11) $$V=\frac{y_{i}\left(w.x_{i}b\right)}{\geq 1-\xi_{i}}$$

where, w is the coefficient vector that defines the hyperplane positioning in the feature space, b is the offset of the hyperplane from the source, and $\xi_{i}$ is the positive loose parameters [189,190]. To achieve optimal hyperplane, an optimization problem is resolved using the following mathematical equation [189,190]:

(12) $$\mathrm{Minimize}\;\sum_{k=0}^{n}\alpha_{i}-\frac{1}{2}\sum_{i=1}^{n}\sum_{j=1}^{n}\alpha_{i}\alpha_{j}y_{i}y_{i}\left(x_{i}x_{j}\right)\;\mathrm{Subject}\;\mathrm{to}\;\sum_{i=1}^{n}\alpha_{i}y_{j}=0,\;\;\;\;0\leq \alpha_{i}\leq C,$$

where $\alpha_{i\;}$ is the Lagrange multiplier, and C is the model penalty. Thus, the decision function is demonstrated as follows:

(13) $$g\left(x\right)=sign\left[\overset{n}{\underset{i=1}{\sum }}y_{i}\alpha_{i}x_{i}+b\right]$$

In an instance where the separating sample is nonlinear, the decision function is otherwise written as:

(14) $$g\left(x\right)=sign\left[\overset{n}{\underset{i=1}{\sum }}y_{i}\alpha_{i}K\left(x_{i},x_{j}\right)+b\right]$$

In this study, the initial data was converted to higher dimensional space by means of kernel functions (*K*). A number of kernel functions are available in SVM (polynomial, linear, sigmoid and radial basis) [190,191]. Prominently and most widely adopted kernel for mass movement applications, the radial basis function (RBF—Equation (15)) [78,101], is used in this study:

(15) $$K\left(x_{i},x_{j}\right)=exp\left(\frac{-∥x_{i}-x_{j}∥{}^{2}}{2\delta^{2}}\right)$$

where $\delta^{2}$ is the bandwidth of the RBF used to adjust the generalization, $x_{i}$ is the i*-th* support vector, and $x_{j}$ is the j*-th* support vector. The hyperplane function can be described as:

(16) $$f\left(x\right)=\omega^{T}x+b$$

where ω  and b are the coefficient.

The critical limitation conditions over an original optimization problem based on the SVR model explained as follows:

(17) $$min\frac{1}{2}\omega^{T}\omega +C\sum_{i-1}^{N}(\xi_{i}+\xi_{i}{}^{*})$$

### 3.4. Model Validation and Evaluation

**Figure 4:**
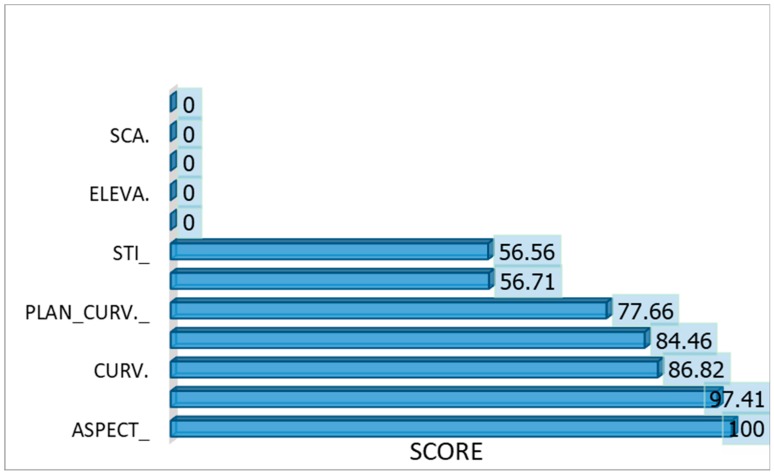
Conditioning factor importance: PROFCUV = Profile curvature, CURV_ = Total Curvature, SLOP_ = Slope angle, PLAN_CURV_ = Plane Curvature, TWI = Topographic Wetness Index, STI = Stream Transportation Index, Roughness = Surface Roughness Index, ELEVA. = Elevation, SCA = Stream Catchment Index, TPI = Topographic Position Index.

To evaluate the debris flow model performance, we used Receiver Operating Characteristics (ROC) sensitivity, specificity and accuracy evaluation techniques [6,104,125,192]. Area Under the Curve (AUC) values were used to compare the success and prediction rates [11,149,193,194]. ROC curve assesses the model’s capability to differentiate between the presence and absence of even represented in the specificity versus sensitivity measures (Figure 4). The ROC curve plots the false positive rate on the X-axis and true positive rate on the Y-axis. A good model has indicated with ROC curve fitted upper-left side and AUC value close to 1. Ordinal association between the model’s success rate is adjudged to be poor (0.5 to 0.6), average (0.6 to 0.7), good (0.7 to 0.8), very good (0.8 to 0.9) or excellent (0.9 to 1.0) [92,195].

Additionally, global performances of the model were evaluated to ascertain the power of the model in terms of prediction and capability strengths presented as success rate and prediction rate respectively; using the ROC/AUC (Equation (9)) [6,11,52,99,107,192,196]. The ROC curve is a dual scale plotted graph of Equation (18) against Equation (19). The curves are plotted on a Cartesian graph, calibrated on both positive axes (y-axis and x-axis) or vertical and horizontal lines with a scale units range from 0 to 1. It has been reported that the closer the curve toward the upper left of the vertical axis, the superior the model performance [99]. The quality of a model prediction or classification is demonstrated in a quantitative AUC value of categories scale as follows:

(18) $$ROC=\frac{Sensitivity}{1-Specificity}$$

(19) $$Specificity=\frac{TN}{TP+TN}$$

(20) $$Sensitivity=\frac{TP}{TN+TP}$$

where TP is the True positive, TN denote the True negative.

## 4. Results

In this study, twelve conditioning factors (Table 1) were prepared and optimized to predict area susceptibility to debris flow in Cameron Highlands. The multi-collinearity effects amongst the twelve conditioning factors and important contribution in the debris flow modelling using MARS rank are shown in Table 1. The Information Value (IV) indicated that profile curvature has an insignificant influence of about 28% on the other variables meaning that, it has no collinearity effect and strongly agreed by Cramer’s values (26%) and Gini coefficient (7%). The second level is the slope aspect with 40% IV, 30% Cramer’s Value and 23% Gini coefficient and is the most important variable in debris flow prediction with 100% MARS score function. RSPI was observed to have the highest multi-collinearity effect under IV of 94%. This is evidently shown on other evaluation metrics, which considered as a bad predictor and discarded from the advance analysis. The Cramer’s V relationship score reveals no significant correlation between each variable, which coincided with [176] this translate to no multi-collinearity effect present in the variables at 95% confidence level. In Gini coefficient, SCA, RSPI, TWI, STI and Profile Curvature demonstrated the presence of multicollinearity effects; this shows a certain level of disagreement, especially with Profile curvature. To satisfy the requirement for feature subset selection that is highly correlated with the target, there is a need for further evaluation on the training data [176].

Further analysis on the contribution of each variable through an optimization process with MARS algorithm indicates that only seven factors significantly influence the outcome. The measure of relative importance ranked in percentage is presented in (Figure 4). The most contributed or important conditioning factor in order of affinity is the slope aspect (100%) scored value and the least is STI (57%). Slope aspect contributes most to debris flow susceptibility with a measure of 100%; possibly because of the monsoon airstream bearing on the topography, which tends to make the high-slope side much more prone to debris flow initiation. Closely following the slope aspect is profile curvature, total curvature, slope angle and plan curvature with 97.41%, 86.82%, 84.46% and 77.66%, respectively. The other relative important variables are topographic wetness index and sediment transport index with 56.71% and 56.56% respectively. Similar feature subset selection was established by Jakob and Horton et al. [71,115]. The remaining five (TRI, elevation, RSPI, SCA and TPI) variables yielded zero or no contribution to the model building despite their theoretical contribution in mass movements modelling.

### 4.1. R-SVM for Debris Flow Susceptibility Mapping

**Figure 5:**
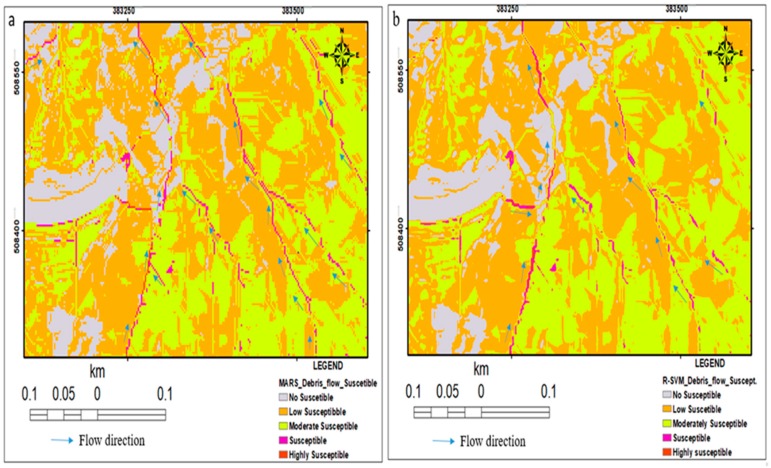
Debris flow susceptibility using: (**a**). Multivariate Adaptive Regression Splines (MARS) model and (**b**). Support Vector Machine-Regression (R-SVM).

R-SVM regression equation was used to generate a continuous debris flow susceptibility index map with a value range from 0.005 to 0.776. The quantitative model prediction index from the pixel values was classified into five classes using natural break classification scheme (0.005–0.155, 0.156–0.305, 0.306–0.455, 0.456–0.605, 0.606–0.776), this index received threshold digital number between 0 and 1 which signifies the possibility of debris flow pixel value in the catchment area. Afterwards, similar to the MARS result, both susceptibility indices maps were reclassified [6,78] into five classes (e.g., No susceptible, Low susceptible, Moderate susceptible, Susceptible and Highly susceptible) using natural break classification scheme and assigned qualitative importance of debris-flow susceptibility pixel value of to the classes (Figure 5a,b). 

### 4.2. Debris Flow Susceptibility Mapping with MARS Approach

In this research, 15 BFs and knot locations generated and shown in Table 2, BF used to describe individual interval of the conditioning factors. The debris flow susceptibility map generated by MARS model was produced using Equation (21) and rendered in Figure 5. This is executed in ArcGIS 10.6 software environment, MARSpline Salford Predictive Modeler (SPM) 8.1 software (https://www.salfordsystems.com); that generated the susceptibility map as shown in Figure 5.

Debris Flow (DF) = 1.17375 + 50.778 × *BF*3 − 59.505 × *BF*14 − 3.07208 × *BF*27 + 11.5545 × *BF*28 + 1.99699 × *BF*30 + 5.81348 × *BF*40 − 1.2703 × *BF*53 + 7.03378 × *BF*108 + 6.16987 × *BF*120 + 29.2086 × *BF*136 + 31.4915 × *BF*138 − 6.84261 × *BF*140 − 5.26128 × *BF*146 + 3.93846 × *BF*147 − 4.98071 × *BF*171. (21)

**Figure 6:**
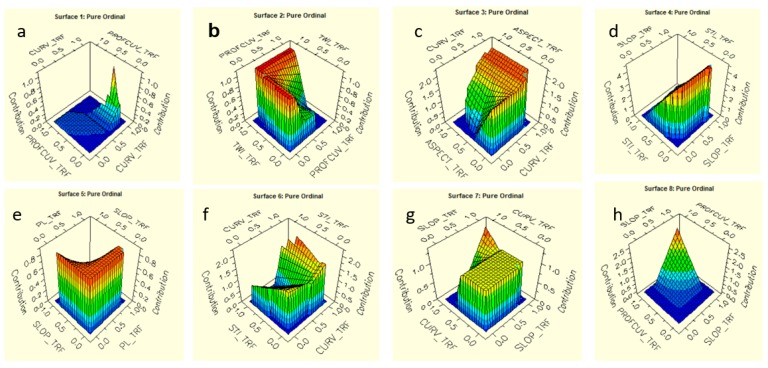
Response surface curve—Relationship between individual independent variable and the contribution of the target variable for (**a**) Total curve and profile curvature, (**b**) TWI and profile curvature, (**c**) Aspect and total curvature, (**d**) STI and slope gradient, (**e**) Slope gradient plan curvature, (**f**) STI and curvature, (**g**) total curvature and Slope gradient, and (**h**) profile curvature and slope angle.

The relationships between the explanatory variables and the contribution of the target variables are demonstrated on the response surface curves (Figure 6a–h). The figure explains the interaction between a pair of variables. Other contributions of the slope aspect, STI in describing the spatial distribution of debris flow shown in Figure 6c,d,f–h. 

The seven optimal conditioning factors subset (Table 1) were utilized as input variables into MARS and R-SVM algorithm that produces the debris flow susceptibility model and generates a regression equation (Equation (20)) for the final debris flow susceptibility map (Figure 5). MARS model outputs susceptibility index value range is from 0.005 to 0.9999. This was further classified into five levels, which include Not Susceptible, Low Susceptible, Moderate Susceptible, Susceptible and Highly Susceptible using equal interval classification method (Figure 5). 

### 4.3. Model Performance Evaluation

**Figure 7:**
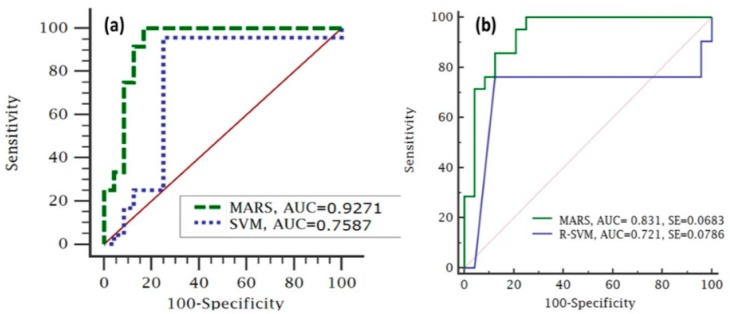
Receiver Operation Characteristic (ROC) comparison of debris flow using MARS and R-SVM: (**a)**. success rate and (**b)**. Prediction rate.

The general quantitatively produced performance accuracies of the debris flow model based on the ROC curve approach is shown in Figure 7a,b success rate and prediction rate by equating the two susceptibility maps with the debris flow training and validation datasets respectively. From the result, we observed that the MARS and R-SVM algorithms showed promising performance for debris flow susceptibility evaluation AUC between 0.72 and 0.92. The plot of the ROC curve Figure 7 and analysis of the indicators provided a quantitative appraisal of the model performance.

Performance evaluation of the two models using ROC curve indices shows that the MARS model transcended R-SVM model with AUC values of 0.9345 and 0.7587 respectively. Using the validation dataset, the model prediction rate produced AUC values of 0.831 and 0.721 for MARS and R-SVM models respectively. MARS have the highest success rate and prediction rates 0.935 and 0.832 respectively. The R-SVM model has the lowest measure of accuracy with a success rate of 0.7587 and prediction rate of 0.721.

## 5. Discussion

Studies such as [11,55,115,160,197,198,199] have confirmed the significance of slope aspect, profile curve, total curve and slope gradient to geo-hazard modelling. They observed the significant contribution of these conditioning factors (slope angle, plan curvature and flow accumulation) to debris flow initiation. In this study, STI, Roughness, Elevation, SPI, SCA and TPI were eliminated from advance analysis because they have zero scores and therefore lack predictive ability. Delmonaco et al. [200] pointed out that sediment availability, water input and slope gradient are the three main factors that directly or indirectly affect the possibility of debris flow initiation. Plan curvature, TWI and STI on the other hand, reveal the proportion of soil water content associated with the soil characteristics and denudations [198,201]. Nevertheless, the TWI is related more to debris flows than to landslides in low-order channels [124,162,202]. A high TWI value suggests the presence of surface depressions that prevent surface runoff and store surface water [48]. The factors excluded during the optimization process (elevation, SPI, surface roughness, SCA and TPI) reduces the objective function of the model (minimizing the error term). The excluded conditioning factors were considered redundancy was not involved in the prepared final modelling input independent variables; however, it does not mean they are not useful in the development of debris flow processes elsewhere. For instance, several studies have highlighted the role of elevation, SCA and SPI in debris flow and landslides in different environments [38,89,90,94,203]. 

The response curves relationship identified the contribution of profile curvature and TWI in debris flow potential modelling, this could be responsible for the hydrological effect on the debris flow catchment area. Thus, refers to an explanatory factor of fluid accumulation at a catchment area and signpost the tendency for debris flow propagation or run-out downslope due to the influence of gravitational forces [77]. Water permeation effects on soil strength divulge and reactivate the flow of debris from slope curvature of water to the lower values of profile curvature, which contributes most. From Figure 6e, slope gradient contributes intensively to the debris flow initiation to deposition at an angle range of 15° to 35° [11,115,173,204] and deposition zones (≤9°).

The quantitative susceptibility indices range from 0.005 to 0.9999 and 0.005 to 0.776 for MARS and R-SVM respectively. A pixel with 0 value indicates no possibility of debris flow while a pixel value of 1 indicates absolute debris flow of 100% possibility. The possible values need to be split into a specific number of classes that construct qualitative susceptibility maps to debris flow [105]. A meaningful decision can be derived from the maps via a nominal representation of the results. These were subjected to five qualitative classes the using natural break scheme, due to its adherence to a specific framework that manipulated the class into non-user defined categories [77]. The catchments were categorized into five areas of susceptibility levels; Not Susceptible, Low Susceptible, Moderate Susceptible, Susceptible and Highly Susceptible. The area in red colour denotes highly debris flow susceptibility. In contrast, visual susceptibility maps showed a glaring conflicting pixel class in Figure 5b, misclassification from highly susceptible class to moderate susceptible class. Drainages and second-order stream were predicted to be highly susceptible area to debris flow as shown in Figure 5a,b; for the purpose of mitigation measure, this zone is strongly not suitable for habitation, hence land-use developers should avoid it.

The model performance reveals MARS approach performing better than R-SVM [120,127,128,129]. The closer the ROC plot is to the upper left corner of the graph, the higher the overall accuracy [79,205,206]. The ROC curves are plotted on a Cartesian graph, calibrated on both positive axes (y-axis and x-axis) or on vertical and horizontal lines with a scale units range from 0 to 1. It has been reported that the closer the curve tends toward the upper left of the vertical axis of the graphs, the superior the model performance [99]. MARS success rate is toward the top right axis, which can term excellent with average prediction rate. 

The competence of simple LiDAR-derived conditioning factor that offers promising and consistent debris flow susceptibility analysis have been demonstrated. This has similarly been proved in flood modelling [77]. However, the model’s efficiencies confirmed the significance of a feature subset selection and variables evaluation at the pre-processing stage of modelling. Although, exploration of advance feature subset selection and other machine learning algorithms could unveil different and better results.

## 6. Conclusions

This research demonstrated the use of laser scanning technology with MARS and R-SVM to develop models for the prediction of an area susceptible to debris flow, in the Ringlet region of the Cameron Highlands, which is affected by recurrent mass-movement related natural disasters. Triggered by anthropogenic and natural factors; utilized appropriate samples of training and test datasets to examine the interplays between the input factors (conditioning factors) and the dependent variable (debris flow inventory) for debris-flow susceptibility mapping. Geomorphometric and topographic elements generated from LiDAR-DEM (slope aspect, profile curvature, slope angle, total curvature plane curvature, TWI and STI) are observable factors that most contributed to debris flow prediction and susceptibility zones mapping in the Cameron Highlands. These parameters can be deemed suitable for a similar form of debris flow studies with minimal processing time and a high accuracy output. 

The debris flow susceptibility maps revealed proficiencies of MARS and R-SVM algorithms with more than 72% AUC accuracy values, which yielded acceptable performance based on the success and prediction rates indicators. Interestingly, the MARS technique performed better with an accuracy difference of 2% in success rate and 1% prediction rates higher than R-SVM; this confirmed the efficacy of MARS application in debris flow prediction and detection of an area susceptible to debris flow. The pixel values generated from the debris flow susceptibility maps were classified into five using Jenk’s natural break classification scheme. The debris flow zone induced by the monsoon rain that twig by landslides will be greater than the events in the absence of landslides and the elevation of the debris source is developed toward the valley or mountain bottom. 

Based on the accomplishments of the models used, this study also highlighted the significant contribution of MARS approach and LiDAR-derived DEM, suggesting for adoption in an area that has similar geographical settings. LiDAR provides high-resolution topographical data that represents the land surface more effectively. Therefore, the extracted topographic parameters from the laser scanning data permit realistic representation of the terrain configuration for debris flow in somewhat more reliable in susceptibility mappings. 

Future investigation will explore the transferability of this model in a different environment using similar temporal LiDAR dataset on diverse topographic characteristics, which can improve model accuracy. In addition, advancement in feature subset selection is needed especially introducing metaheuristic nature-inspired optimization algorithm with Unmanned Aerial Vehicle (UAV) data may produce better results in future.

The outcome of this research paper will contribute to identifying the area susceptible to debris flow for the purpose of significant regional planning, land use and policies making processes. Such strategies can assist the government and decision-makers to reduce the risks associated with debris-flow disaster. 

## Figures and Tables

**Table 1:** Features Selection measure of correlation and importance.

S/N	Variable	Information Value (IV)	Cramer’s V	Gini Coefficient	Important Score
1.	Slope Aspect	0.40	0.30	0.23	100
2.	Profile Curvature	0.28	0.26	0.07	97.41
3.	Total curvature	0.80	0.41	0.17	86.82
4.	Slope angle	0.62	0.37	0.06	84.46
5.	Plan curvature	0.57	0.36	0.15	77.66
6.	TWI	0.88	0.44	0.02	56.71
7.	STI	0.48	0.32	0.03	56.56
8.	TRI	0.68	0.47	0.06	0.00
9.	Elevation	0.61	0.36	0.33	0.00
10.	RSPI	0.94	0.45	0.03	0.00
11.	SCA	0.52	0.34	0.00	0.00
12.	TPI	0.49	0.30	0.01	0.00

**Table 2:** BFs and knot locations.

No.	Relationship
1.	*B*F3 = *max* (0, *CURV_TRF* − 0.868835) × *BF1*;
2.	*BF*14 = *max* (0, *CURV_TRF* − 0.567935);
3.	*BF*27 = *max* (0, 0.852459 − *TWI_TRF*) × *BF1*;
4.	*BF*28 = *max* (0, *ASPECT_TRF* − 0.539546) × *BF*15;
5.	*BF*30 = *max* (0, *STI_TRF* − 0.5587) × *BF*24;
6.	*BF*40 = *max* (0, *PROFCUV_TRF* − 0.36921) × *BF6*;
7.	*BF*53 = *max* (0, *PL_TRF* − 0) × *BF24*;
8.	*BF*108 = *max* (0, *STI_TRF* − 0) × *BF15*;
9.	*BF*120 = *max* (0, *CURV_TRF* − 0) × *BF18*;
10.	*BF*136 = *max* (0, *CURV_TRF* − 0.554817);
11.	*BF*138 = *max* (0, *CURV_TRF* − 0.582064);
12.	*BF*140 = *max* (0, *ASPECT_TRF* − 2.98023 × 10^−8^) × *BF139*;
13.	*BF*146 = *max* (0, 0.977528 − *SLOP_TRF*) × *BF138*;
14.	*BF*147 = *max* (0, *PROFCUV_TRF* − 0.188011) × *BF24*;
15.	*BF*171 = *max* (0, *STI_TRF* − 0.0797712)

Notes: PROFCUV (Profile curvature), CURV_(Total Curvature), SLOP_(Slope), PLAN_CURV_ (Plane Curvature), TWI (Topographic Wetness Index), STI (Stream Transportation Index), Roughness (Surface Roughness Index), ELEVA (Elevation), SCA (Stream Catchment Index) and TPI (Topographic Position Index).
